# Unconsolidated Fracture of the Thigh Successfully Treated by Acupuncture

**Published:** 1851-11

**Authors:** 


*Unconsolidated Fracture of the Thigh successfully treated by Acupuncture.* By M. Lenoir.—The rationale of the various plans of treatment which have been adopted, in order to prevent the formation of false joints, consists in the establishment of an inflammatory action in the fibrous tissue, situated between the bony fragments, and the consequent secretion of a secondary callus. One of the methods proposed has, in the hands of its inventor, M. Malgaigne, been unattended with success: we mean acupuncture. But the following case, communicated to the Societié de Chirurgie, by M. Lenoir, proves that this mode of treatment deserves some notice, even although it has not afforded similar results to M. Maisonneuvre. Much of the success obtained by M. Lenoir must, doubtless, be attributed to the many precautions observed by him.

Dupèchè, a carpenter by trade, aged thirty-three years, in falling from a height of fifty-two feet, fractured his right thigh. He was immediately conveyed to La Pitié, and placed under the care of M. A. Bérard. After fifty-four days of treatment, the patient began to walk with the assistance of crutches, when M. A. Bérard, in order to remove the stiffness which existed in the knee-joint, endeavored by force to extend the motions of this articulation; in one of these manœuvres the neck of the femur gave way, and the signs of the fracture re-appeared. The broken bone was again reduced, and an immovable apparatus applied to keep the fractured ends *in situ*; at the termination of a month the apparatus was removed, but the fracture had not consolidated, and the patient had himself conveyed home.

Six months afterwards, M. Lenoir took him into the hospital for the purpose of employing the treatment by acupuncture; but before trying this plan, he used all the means likely to insure success, and, amongst others, he had him placed on a mechanical bed, so as to maintain complete freedom from motion, even in attending to the calls of nature. As the fracture was oblique, and the upper fragment very sharply bevelled, and the fragments, by overlapping, occasioned a shortening of about two and a half inches, M. Lenoir had an apparatus, for maintaining extension, constructed by a carpenter, a friend of the patient. This apparatus consisted of a long box, nearly in the shape of the limb, and consequently wider above than below, but longer than it; it was about three inches deep, and was composed of three pieces of light wood closely united to one another; of these three splints, the external was eight inches longer than the others, which terminated at the junction of the thigh with the trunk; this longer portion had at its upper end a mortise intended to facilitate the employment of counter extension; to the lower end of this groove a kind of toothed wheel and axel was adapted, to which was applied a catch for the purpose of fixing it. This apparatus, lined with carded cotton, received the limb, the foot being covered with a gaiter of ticken furnished with a foot-strap; by means of this strap rolled round the wheel, extension was maintained by another strap well padded, passing along the fold of the groin, having the ischium as its *point d' appui*, and its ends fixed in the mortise in the outer splint of wood.

For several days nothing was done except to tighten the straps according as they became relaxed. At last, on the 12th of August, seven months and some days after the accident, M. Lenoir proceeded to insert the needles. At first he introduced four, each being four inches long, and furnished with a head. Their points were directed along the inner surface of the upper fragment, from below upwards; an interval of but half an inch being left between each needle. Contrary to his expectation, and although he passed them in as far as the heads, he met no obstacle to their introduction. This, doubtless, depended on the existence of an interval between the two fragments, the extension effected by the apparatus having reduced the fracture only in the direction of the length of the limb, and not transversely. The four needles remained *in situ* for six days; at first they excited redness of the skin, then a little pus appeared about them and rendered them moveable, and finally, a slight swelling occurred. These symptoms indicating that inflammation had developed itself, M. Lenoir withdrew the four needles; and, after having cleaned them, he re-introduced them higher up, following carefully the direction of the upper fragment, and leaving between them the same intervals as before. The same symptoms followed this second operation; at the end of five days the needles had become moveable, and were taken away; and the inflammatory action now appearing to be sufficient to produce union, the introduction of the needles was not repeated. The inflammatory swelling of the limb was treated by poultices, antiphlogistic diet, and cooling drinks; and when it was subdued, the two surfaces of the fragments were brought into closer proximity by means of small splints placed around the thigh, and tightened by two straps of leather, a practice previously employed by Amesbury. The apparatus was inspected daily, and tightened when necessary. At the end of twenty-three days, in order to ascertain how far consolidation had advanced, the limb was completely uncovered; it was found to have neither got out of shape nor undergone retraction; but when the hand was passed over the seat of the fracture, it still yielded; splints were immediately re-applied, the limb was replaced in its groove, and extension continued. No fresh examination was made until the expiration of thirty-five days from this time, and then the callus was found to be sufficiently solid to justify the removal of the entire apparatus. Carefully measured, the limb was found to be rather less than eight-tenths of an inch shorter than that of the opposite side; the knee-joint was stiff, but the patella was still capable of transverse motion; the thigh and upper part of the leg were œdematous, but otherwise there was no apparent deformity at the seat of the fracture, and the callus was not very bulky. Lastly, the coxo-femoral articulation was capable of motion, and the patient was able to raise the limb by the unaided action of the muscles. As an additional security, he was advised to keep his bed, and, during a fortnight that he was confined to it, the œdematous swelling of the limb was treated by fomentations of aromatic wine, and by bandaging. At the end of that time he got up and walked, at first with the aid of crutches, and afterwards of a single stick; finally, he left the hospital cured, and M. Lenoir subsequently ascertained that on his return to his native district (Auvergne,) he had, during the entire following autumn, driven a plough, and that he now experiences no difficulty in the pursuit of field labor.—*Dublin Quar. Journal, from Bulletin Général de Thérapeutique*, Dec. 15, 1850.

